# Long-Term Vonoprazan and Acotiamide-Refractory Patients With Functional Dyspepsia Partly Exhibit Pancreatic Enzyme Abnormalities

**DOI:** 10.7759/cureus.70371

**Published:** 2024-09-28

**Authors:** Ken Nakamura, Seiji Futagami, Shuhei Agawa, Sakura Higashida, Takeshi Onda, Rie Kawawa, Mayu Habiro, Nobue Ueki, Katsuhiko Iwakiri

**Affiliations:** 1 Department of Gastroenterology, Nippon Medical School, Tokyo, JPN

**Keywords:** acotiamide, duodenum, functional dyspepsia, pancreatic enzyme abnormalities, vonoprazan

## Abstract

Background: Although a new potassium-competitive acid blocker (P-CAB) vonoprazan has been developed in Japan, no data are available regarding long-term vonoprazan or vonoprazan and acotiamide combination treatment in patients with functional dyspepsia (FD).

Methodology: A total of 73 consecutive patients with FD diagnosed according to the Rome III classification were enrolled. Forty-two patients with FD were treated with vonoprazan monotherapy and thirty-one patients with FD were treated with vonoprazan and acotiamide combination therapy for 24 weeks. The levels of five pancreatic enzymes were measured, and the overall treatment efficacy (OTE) was defined as the ratio of FD patients with improved or unchanged in all items of GSRS and FD symptom scores after the treatment.

Results: Treatment with vonoprazan monotherapy and vonoprazan and acotiamide combination therapy significantly improved FD symptoms. There were no significant differences in OTE between patients treated with vonoprazan monotherapy (42.9%) and those treated with vonoprazan and acotiamide combination therapy (52%). There were no significant differences in duodenal eosinophilic infiltration between the improved and unimproved groups treated with vonoprazan alone and vonoprazan and acotiamide combination therapy, respectively. In contrast, there was a significant difference (*P* = 0.004) in the ratio of pancreatic enzyme abnormalities between the improved and unimproved patients treated with vonoprazan monotherapy and those treated with vonoprazan and acotiamide combination therapy.

Conclusions: Long-term vonoprazan alone or vonoprazan and acotiamide combination therapy significantly improved each FD symptom. The OTE in patients treated with vonoprazan alone or vonoprazan and acotiamide combination therapy was only 50%. Long-term vonoprazan and acotiamide combination therapy may differentiate patients with pancreatic enzyme abnormalities from those with FD.

## Introduction

Functional dyspepsia (FD) is a global concern that affects numerous individuals. Due to the complicated pathogenesis of FD, definitive treatments have not yet been established. Acotamide, a novel gastrointestinal (GI) motility modulator, was developed in Japan in 2013. The Cochrane database of systemic reviews indicated that proton pump inhibitors (PPIs) are suitable for the treatment of patients with FD [1]. Previous studies have reported that patients with PPI therapy-resistant FD experience abdominal discomfort or indigestion, with a prevalence of approximately 20% in the general population [2]. Then, PPI and acotiamide combination therapy was highly effective in improving PPI-refractory FD symptoms [3]. Recently, a new potassium-competitive acid blocker (PCAB), vonoprazan, has been widely used in Japan [2]. A Japanese retrospective study reported an improvement rate of 59%, in patients with FD, after four weeks of vonoprazan therapy (20 mg) [2].

Although the symptoms of patients with exocrine pancreatic dysfunction reportedly overlap with those of dyspepsia [4,5] and several studies have demonstrated abnormal pancreatic function in patients with dyspepsia [6-8], there are no available data to explain the direct relationships or linkages between pancreatic enzyme abnormalities and dyspeptic symptoms. We previously reported that pancreatic enzyme abnormalities occur concomitantly in patients with refractory FD [9]. Furthermore, we previously attempted to divide the characteristics of patients with FD alone from those with pancreatic enzyme abnormalities (FD-P) as refractory FD and found no significant differences in the clinical characteristics of patients with FD and FD-P [10]. Therefore, patients with PPI therapy-resistant FD are partly regarded as having pancreatic diseases.

In this study, we aimed to determine whether long-term vonoprazan monotherapy or vonoprazan and acotiamide combination therapy for 24 weeks improves the clinical symptoms in patients with FD. In addition, we attempted to clarify whether patients with vonoprazan and acotiamide-resistant FD have any clinical characteristics, the grade of gastric atrophy, duodenal eosinophils infiltration, or pancreatic enzyme abnormalities to clarify the precise pathophysiology in patients with refractory FD.

## Materials and methods

Patients

**Figure 1:**
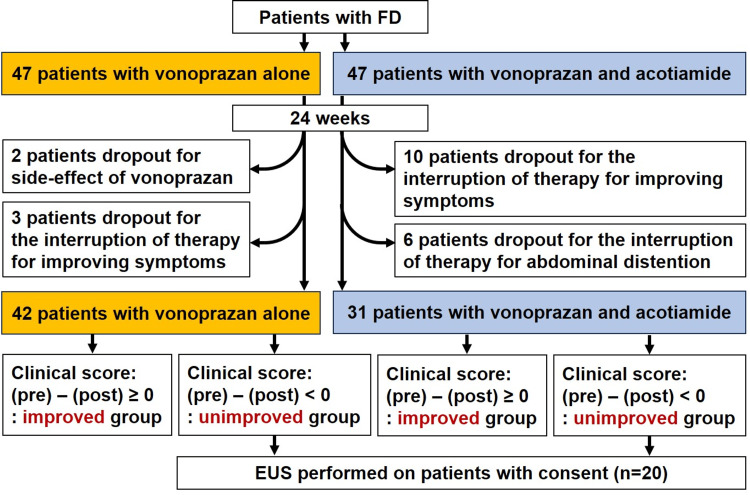
Study protocol. This study is a prospective clinical study. We divided two groups (vonoprazan monotherapy or vonoprazan and acotiamide combination therapy) in a blinded manner. Although 94 patients with FD were enrolled, five FD patients with vonoprazan alone were omitted and 16 FD patients with vonoprazan and acotiamide combination therapy were omitted in 24 weeks. Image credit: Seiji Futagami. EUS, endoscopic ultrasound; FD, functional dyspepsia

Patients diagnosed with FD after upper GI endoscopy, abdominal ultrasonography (US), and abdominal computed tomography (CT) between August 1, 2021, and September 30, 2023, were recruited from Nippon Medical School Musashi Kosugi Hospital and Nippon Medical School Hospital. Patients were diagnosed with FD based on the absence of abnormal findings related to FD symptoms on imaging (upper GI endoscopy, abdominal US, and abdominal CT). Patients with FD were diagnosed based on the Rome III criteria [11]. This study is prospectively clinical. Eligible patients were randomly divided into two groups (vonoprazan monotherapy or vonoprazan and acotiamide combination therapy) (Figure 1). Although 94 patients with FD were gotten written informed consent and registered, five FD patients with vonoprazan alone were omitted and 16 FD patients with vonoprazan and acotiamide combination therapy were omitted in 24 weeks (Figure 1). A total of 73 consecutive patients were included in this study. Forty-two patients with FD were administered vonoprazan alone and 31 patients with FD were administered vonoprazan and acotiamide combination therapy for 24 weeks (Figure 1). Vonoprazan alone or vonoprazan and acotiamide combination therapy was determined in a blinded manner. The exclusion criteria were severe heart disease, renal or pulmonary failure, liver cirrhosis, severe systemic illness, psychiatric disorders, psychotropic drug intakes, and a history of malignant diseases. We measured p-amylase, lipase, trypsin, phospholipase A2 (PLA2), and elastase-1 levels in the sera of the enrolled patients. The study protocol was approved by the Ethics Review Committee (758-5-68) of the Nippon Medical School Hospital on March 2, 2021. Written informed consent was obtained from the participants.

Definition of pancreatic enzyme abnormalities

Serum trypsin, PLA2, lipase, p-amylase, and elastase-1 levels were measured using an automated chemistry analyzer (AU 5822 Analyzer; Beckman Coulter, Brea, CA). Pancreatic enzyme abnormalities were defined as the absence of abnormal imaging findings and the presence of any pancreatic enzyme abnormalities, including those of p-amylase, lipase, trypsin, PLA2, and elastase-1. The validated reference range of pancreatic enzymes at our hospital is 100-550 for trypsin, 130-440 for PLA2, 11-53 for lipase, 18-53 for p-amylase, and 0-300 for elastase-1.

Clinical symptoms

FD was diagnosed based on the Rome III criteria [11]. Postprandial distress syndrome (PDS), epigastric pain syndrome (EPS), and EPS overlapped with PDS (EPS&PDS) were defined when symptoms persisted for at least three months and the onset of symptoms occurred at least six months before the diagnosis. In this study, FD symptoms and treatment satisfaction were evaluated based on the Rome III classification as follows: 0, none; 1, very mild; 2, mild; 3, moderate; 4, severe; and 5, very severe [11]. Clinical symptoms were also estimated pre- and post-treatment using the Gastrointestinal Symptom Rating Scale (GSRS) [12]. We used the mean GSRS score and associated 15 GI symptoms for the evaluation of dyspeptic symptoms. Clinical scores in patients with pretreatment compared with those in posttreatment were more than 0 or 0 was defined as the improved group. Overall treatment efficacy (OTE) means the ratio of FD patients with improved or unchanged in all items of GSRS and FD score.

Endosonographic assessment

An Olympus endosonography device (GF-UCT 260, Olympus, Tokyo, Japan) was used to perform EUS under conscious sedation in 20 patients with treatment-resistant FD after the informed consent (Figure 1). Endosonographic findings were defined as described in a previous study [13]. The endsonographic score (from 0 to 4) was estimated as the sum of the EUS findings. Four clinical criteria, including epigastric pain and the presence of more than two features of EUS, are needed for the determination of early chronic pancreatitis (ECP) [13]. ECP was diagnosed based on the imaging findings of two or more of the four EUS images: (1) hyperechoic foci or strands, (2) lobularity, (3) hyperechoic main pancreatic duct (MPD) margin, and (4) dilated side branches and clinical indications of two or more symptoms, abnormalities in blood/urine pancreatic enzymes, exocrine pancreatic dysfunction, and chronic alcohol intake (60 g/day) [13]. Patients with FD-P were distinguished from those with ECP based on the EUS findings.

Estimation of duodenal eosinophils infiltration and gastric atrophy

We evaluated duodenal eosinophils infiltration as the number of eosinophils using hematoxylin and eosin (HE)-stained specimens from the duodenal second part. The mean value of duodenal eosinophils (in four fields) was determined by two experienced pathologists in a blinded manner. The grade of gastric atrophy was evaluated (from 0 to 6) based on the Kimura-Takemoto classification as follows: C-0: 0, C-I: 1, C-II: 2, C-III: 3, O-I: 4, O-II: 5, and O-III: 6.

Statistical analysis

Two-tailed unpaired t-test was used for continuous variables, and Pearson's chi-square test was used for categorical variables. Statistical analyses were performed using SPSS (version 27.0, IBM Corp., Armonk, NY), and statistical significance was set at *P* < 0.05.

## Results

Comparison of clinical characteristics of enrolled patients

We first compared the clinical characteristics of patients treated with vonoprazan alone with those treated with vonoprazan and acotiamide combination therapy. There were no significant differences in age, gender, grade of gastric atrophy, or FD subtypes between the two groups (Table 1). There were no significant differences in the pancreatic enzyme levels between vonoprazan alone and vonoprazan and acotiamide combination therapy (Table 1).

**Table 1:** Clinical characteristics between vonoprazan alone and vonoprazan and acotiamide combination. Two-tailed unpaired t-test was used for continuous variables (no symbols), Pearson's chi-square test was used for categorical variables (†). F, female; M, male; FD, functional dyspepsia; EPS, epigastric pain syndrome; PDS, postprandial distress syndrome; EPS&PDS, EPS overlapped with PDS; p-AMY, p-amylase; PLA2, phospholipase A2

Factors	Vonoprazan alone (*n* = 42)	Vonoprazan and acotiamide combination (*n *= 31)	*P-*value
Age (mean ± SD)	56.3 ± 3.3	49.8 ± 3.8	0.225
Gender (F/M)	25/17	23/8	0.360
Gastric mucosal atrophy (Closed type/Open type)†	33/9	27/4	0.493
FD score (mean ± SD)	9.02 ± 0.45	8.81 ± 0.45	0.754
EPS/PDS/EPS&PDS†	20/7/15	12/12/7	0.141
p-AMY (U/L)	36.7 ± 2.3	36.0 ± 1.8	0.811
Lipase (U/L)	37.7 ± 4.4	36.3 ± 2.8	0.807
Trypsin (ng/mL)	543 ± 59	454 ± 26	0.225
PLA2 (ng/dL)	302 ± 20	287 ± 17	0.591
Elastase-1 (ng/dL)	135 ± 18	129 ± 11	0.796

Improvement of FD symptoms and GSRS scores of patients treated with vonoprazan alone

The scores for epigastric pain, epigastric burning, bothersome postprandial fullness, and early satiety were significantly higher (*P *< 0.001, *P* < 0.001, *P* < 0.001, and *P *= 0.022, respectively) in patients treated with vonoprazan alone than in those who underwent pretreatment (Table 2). The scores for gastroesophageal reflux, abdominal pain, and indigestion, in patients treated with vonoprazan alone, were significantly (*P* < 0.001, *P* < 0.001, and *P* < 0.001, respectively) lower than those in patients with pretreatment (Table 2).

**Table 2:** FD symptoms and GSRS of patients treated with vonoprazan alone. Values are mean ± SD. *P*-values were calculated by the two-tailed unpaired t-test. FD, functional dyspepsia; GSRS, Gastrointestinal Symptom Rating Scale

Clinical symptoms	Pre-treatment	Post-treatment	*P*-value
Epigastric pain	2.85 ± 0.21	1.46 ± 0.21	<0.001
Epigastric burning	1.95 ± 0.23	0.98 ± 0.19	<0.001
Postprandial fullness	2.34 ± 0.21	1.44 ± 0.18	<0.001
Early satiety	1.88 ± 0.20	1.44 ± 0.20	0.022
Gastroesophageal reflux	1.90 ± 0.24	0.83 ± 0.16	<0.001
Abdominal pain	2.78 ± 0.24	1.27 ± 0.21	<0.001
Indigestion	2.46 ± 0.26	1.63 ± 0.20	<0.001
Diarrhea	0.49 ± 0.17	0.44 ± 0.13	0.728
Constipation	0.73 ± 0.18	0.54 ± 0.14	0.243

Improvement of FD symptoms and GSRS scores in patients treated with vonoprazan and acotiamide

The scores for epigastric pain, epigastric burning, bothersome postprandial fullness, and early satiety in patients treated with vonoprazan and acotiamide were significantly lower (*P *< 0.001, *P *= 0.012, *P *= 0.001, and *P *= 0.008, respectively) than those before treatment (Table 3). The scores for gastroesophageal reflux, abdominal pain, and indigestion in patients treated with vonoprazan and acotiamide were significantly lower (*P *= 0.05, *P* = 0.021, and *P* = 0.021, respectively) than those before treatment (Table 3).

**Table 3:** FD symptoms and GSRS of patients treated with vonoprazan and acotiamide. Values are mean ± SD. *P*-values were calculated by the two-tailed unpaired t-test. FD, functional dyspepsia; GSRS, Gastrointestinal Symptom Rating Scale

Clinical symptoms	Pre-treatment	Post-treatment	*P-*value
Epigastric pain	2.85 ± 0.29	1.59 ± 0.26	<0.001
Epigastric burning	1.70 ± 0.29	0.89 ± 0.18	0.012
Postprandial fullness	2.19 ± 0.29	1.33 ± 0.23	0.001
Early satiety	2.07 ± 0.28	1.52 ± 0.25	0.008
Gastroesophageal reflux	1.82 ± 0.31	0.96 ± 0.15	0.005
Abdominal pain	2.15 ± 0.37	1.22 ± 0.21	0.021
Indigestion	2.22 ± 0.33	1.59 ± 0.26	0.021
Diarrhea	0.44 ± 0.19	0.37 ± 0.17	0.602
Constipation	0.82 ± 0.26	0.74 ± 0.22	0.663

Comparison of overall treatment efficacy of patients treated with vonoprazan alone and with vonoprazan and acotiamide

**Figure 2:**
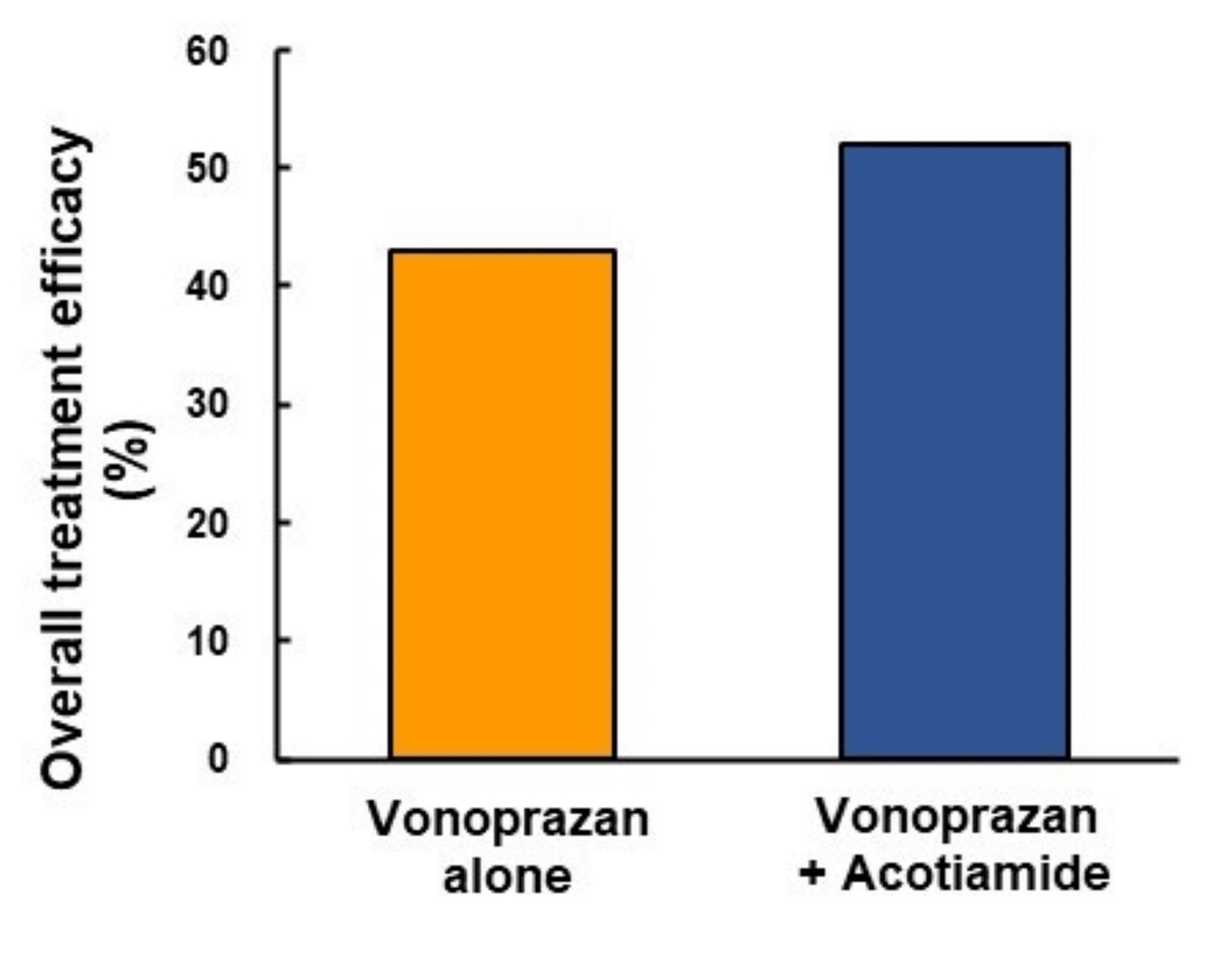
Comparison of overall treatment efficacy between patients who underwent vonoprazan monotherapy and vonoprazan and acotiamide combination therapy. There were no significant differences in the overall treatment efficacy between vonoprazan monotherapy and vonoprazan and acotiamide combination therapy. OTE means numbers of FD patients (pre-pro > 0; all items of GSRS and FD score)/numbers of enrolled FD patients. FD, functional dyspepsia; GSRS, Gastrointestinal Symptom Rating Scale

There was no significant difference in the overall treatment efficacy between the patients treated with vonoprazan alone (42.9%) and those treated with vonoprazan and acotiamide (52%) (Figure 2).

Comparison of duodenal and peripheral eosinophils infiltration between improved and unimproved patients treated with vonoprazan alone or vonoprazan and acotiamide combination therapy

**Figure 3:**
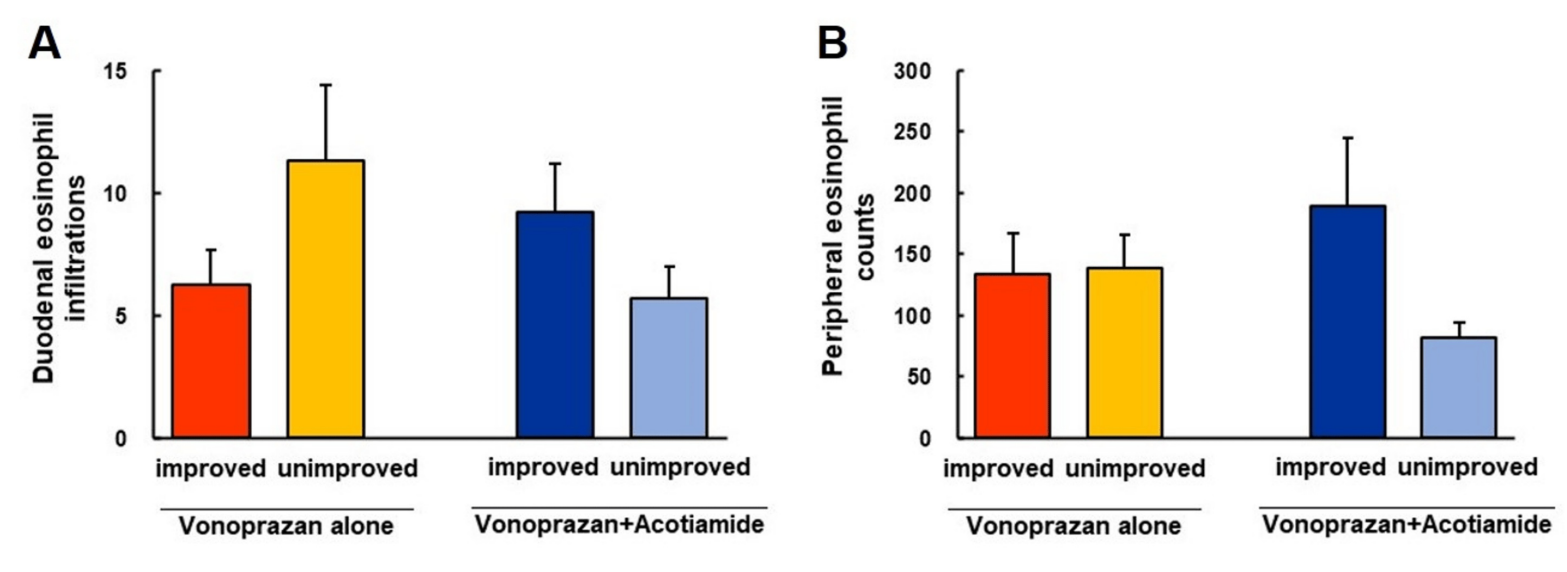
Comparison of duodenal and peripheral eosinophils infiltration between improved and unimproved patients treated with vonoprazan monotherapy and vonoprazan and acotiamide combination therapy (A) There were no significant differences in the duodenal eosinophils infiltration between the improved and unimproved groups treated with vonoprazan alone and vonoprazan and acotiamide combination therapy. (B) There were no significant differences in peripheral eosinophils count between the improved group and unimproved groups treated with vonoprazan alone and vonoprazan and acotiamide combination therapy.

There were no significant differences (*P *= 0.226) in the duodenal eosinophil infiltration between the improved and unimproved groups treated with vonoprazan alone (Figure 3A).

Furthermore, no significant differences (*P *= 0.179) were observed in duodenal eosinophil infiltration between the improved and unimproved groups treated with vonoprazan and acotiamide (Figure 3A). Additionally, there were no significant differences (*P *= 0.925) in peripheral eosinophil infiltration between the improved and unimproved groups treated with vonoprazan alone (Figure 3B). Moreover, no significant difference (*P *= 0.093) in peripheral eosinophil infiltration was observed between the improved and unimproved groups treated with vonoprazan and acotiamide (Figure 3B).

Comparison of the ratio of pancreatic enzyme abnormalities between improved and unimproved patients

**Figure 4:**
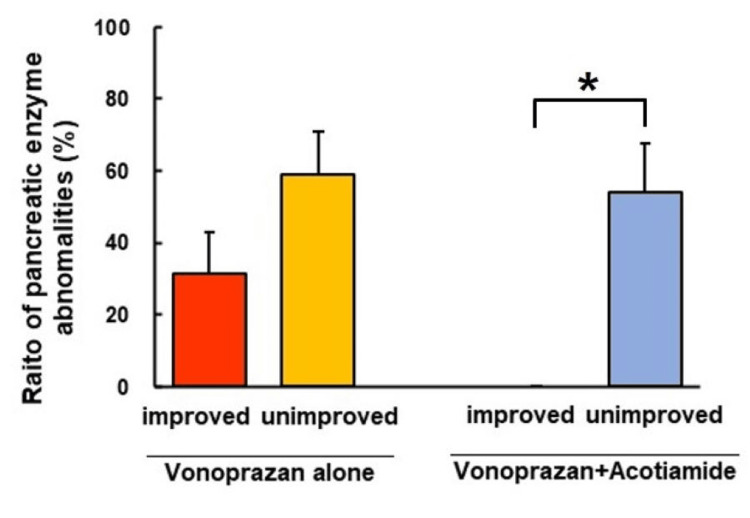
Comparison of the ratio of pancreatic enzyme abnormalities between improved and unimproved patients. There was a significant difference (*P *= 0.004) in the ratio of pancreatic enzyme abnormalities between the improved and unimproved patients treated with vonoprazan and acotiamide. In contrast, there was no significant difference in the ratio of pancreatic enzyme abnormalities between the improved and unimproved patients treated with vonoprazan alone. The ratio of pancreatic enzyme abnormalities means the ratio of functional dyspepsia (FD) patients with any abnormal pancreatic enzyme value. ^*^vs, improved patients with vonoprazan and acotamide combination therapy; *P *= 0.004.

There was a significant difference (*P *= 0.004) in the ratio of pancreatic enzyme abnormalities between improved and unimproved patients treated with vonoprazan and acotiamide combination therapy (Figure 4). In contrast, there was no significant difference in the ratio of pancreatic enzyme abnormalities between improved and unimproved patients treated with vonoprazan alone (Figure 4). There was a significant difference (*P *= 0.004) in the ratio of abnormality of trypsin level between improved and unimproved patients treated with vonoprazan and acotiamide combination therapy (Table 4).

**Table 4:** Comparison of the ratio of pancreatic enzyme abnormalities between improved and unimproved patients treated with vonoprazan monotherapy and vonoprazan and acotiamide combination therapy. *P*-values were calculated by Pearson's chi-square test. p-AMY, p-amylase; PLA2, phospholipase A2

Pancreatic enzymes	Vonoprazan alone		Vonoprazan and acotiamide combination		Vonoprazan alone	Vonoprazan and acotiamide combination
	Improved	Unimproved	Improved	Unimproved	*P*-value	*P*-value
p-AMY (%)	12.5	11.8	0.0	7.7	0.948	0.327
Lipase (%)	18.8	5.9	0.0	15.4	0.258	0.157
Trypsin (%)	25.0	52.9	0.0	53.8	0.101	0.004
PLA2 (%)	25.0	11.8	0.0	15.4	0.325	0.174
Elastase-1 (%)	6.3	0.0	0.0	0.0	0.295	-

Comparison of the score for gastric atrophy between improved and unimproved patients

There were no significant differences in gastric atrophy scores between the improved and unimproved group of patients treated with vonoprazan alone (data not shown). Similarly, there were no significant differences in gastric atrophy scores between the improved and unimproved patients treated with vonoprazan and acotiamide combination therapy (data not shown).

Comparison of endosonographic scores of the unimproved patients who underwent vonoprazan alone and vonoprazan and acotiamide combination therapy

**Figure 5:**
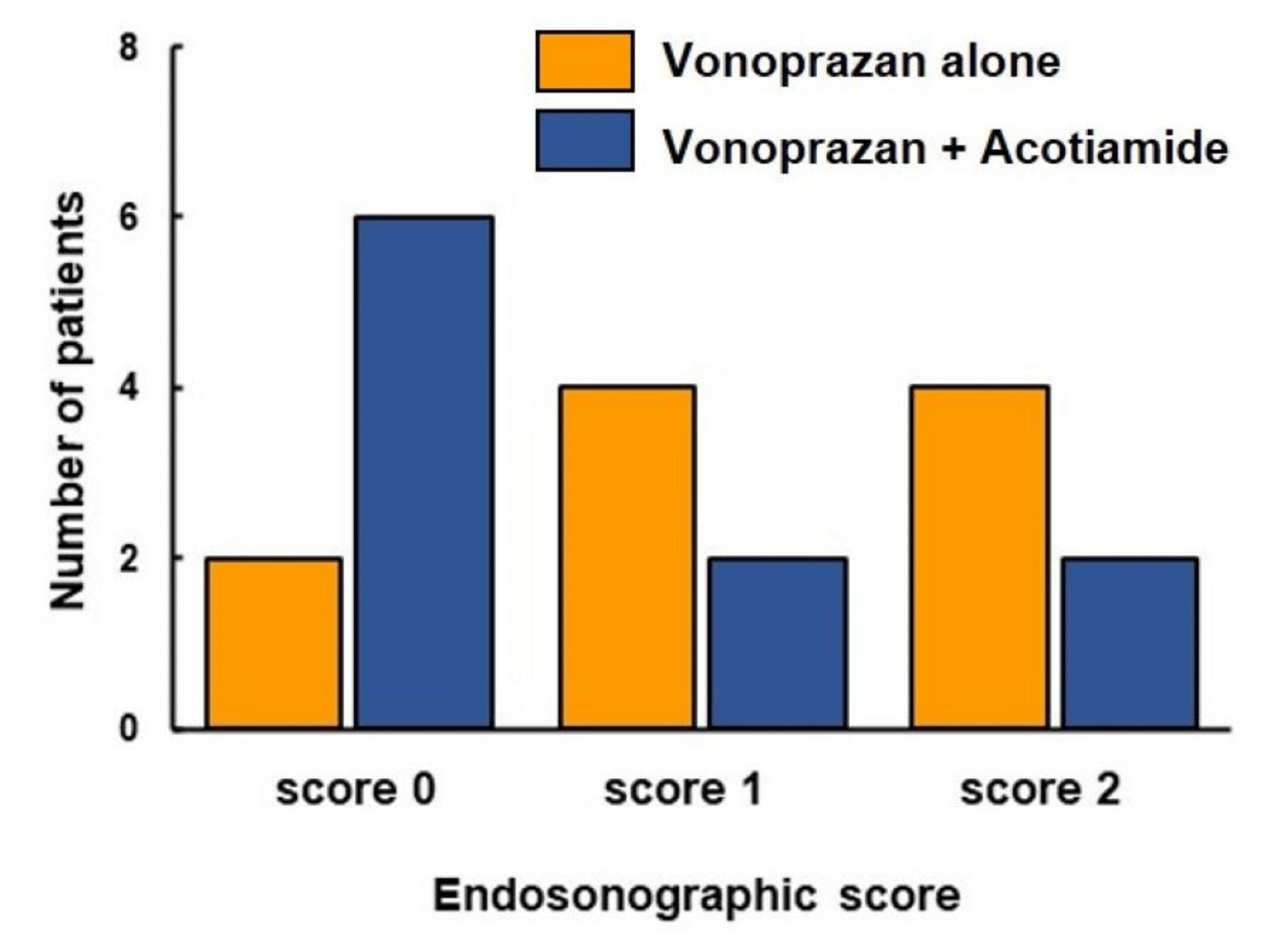
Comparison of score of endosonography between improved and unimproved patients treated with vonoprazan alone and vonoprazan and acotiamide combination therapy. There were two patients in score 0, four patients in score 1, and four patients in score 2 in vonoprazan alone group. There were six patients in score 0, two patients in score1, and two patents in score 2 in vonoprazan and acotiamide combination group.

To clarify whether any endosonographic features differed among the unimproved patients treated with vonoprazan alone or a combination of vonoprazan and acotiamide, we compared the endosonographic scores in unimproved patients who received either therapy. In the vonoprazan monotherapy group, two patients had a score of 0, four patients had a score of 1, and four patients had a score of 2. Six patients had a score of 0, two patients had a score of 1, and two patients had a score of 2 in the vonoprazan and acotiamide combination group (Figure 5).

## Discussion

The main findings of this study were as follows: (1) long-term vonoprazan alone or vonoprazan and acotiamide combination therapy significantly improved each FD symptom. (2)There were no significant differences in the overall treatment efficacy between patients treated with vonoprazan alone (42.9%) and those treated with vonoprazan and acotiamide combination therapy (52%). (3) There was a significant difference (*P *= 0.004) in the ratio of pancreatic enzyme abnormalities between improved and unimproved patients treated with vonoprazan and acotiamide combination therapy.

This study is the first to report the efficacy of long-term treatment with vonoprazan alone or in combination therapy with acotiamide in improving the symptoms of patients with FD. In our study, vonoprazan alone and vonoprazan and acotiamide combination therapy significantly improved each FD symptom, such as epigastric pain, epigastric burning, bothersome postprandial fullness, and early satiety, as described in Tables 2-3. However, the overall treatment efficacy in patients treated with vonoprazan alone or vonoprazan and acotiamide combination therapy was only 50%. Asaoka et al. reported that patients with FD treated with vonoprazan for four weeks had a relatively high response rate (58.8%). We also noted that long-term vonoprazan treatment improved clinical symptoms by almost 50% and that long-term vonoprazan and acotiamide combination therapy improved clinical symptoms, similar to vonoprazan alone. Although we investigated the factors associated with vonoprazan and acotiamide treatment resistance, the expected degree of gastric atrophy and duodenal eosinophil infiltration was not significantly related to patients with vonoprazan and acotiamide combination therapy-resistant FD. In this study, we report, for the first time that vonoprazan- and acotiamide-resistant FD are significantly associated with pancreatic enzyme abnormalities.

Duodenal inflammation has also been associated with the pathophysiology of FD [14-16]. Vanheel et al. demonstrated that not only eosinophils but also mast cells, were increased in the duodenum of patients with FD.17 Duodenal inflammation was also considered a major cause of FD [17]. Considering that no significant differences were found in the duodenal eosinophil count in pretreatment status between the improved and unimproved groups, the possibilities were that duodenal mucosal changes were caused by various factors such as increased mucosal eosinophils and mast cells induced by gastric acidity, bile salts [18], or stress [19], the reduction of gastric acidity, and the alternation of microbiota by long-term vonoprazan treatment as well as PPI [20] may be affected with duodenal eosinophils infiltrations in posttreatment status. Further studies are needed to clarify whether duodenal eosinophil infiltration in posttreatment is associated with clinical improvement in patients with vonoprazan- and acotiamide-resistant FD.

Pancreatic enzyme abnormalities will be associated with resistance to vonoprazan and acotiamide combination therapy. Since previous studies [21,22] have reported that disturbances in the autonomic nervous system may partly affect pancreatic enzyme abnormalities and cause exocrine pancreatic dysfunction, vonoprazan- and acotiamide-resistant FD may involve patients with pancreatic enzyme abnormalities through the disturbances of autonomic nerves. We performed endosonography in 10 patients with FD resistant to vonoprazan monotherapy and in 10 patients with FD who were resistant to combination therapy. The EUS score was zero for six patients with vonoprazan- and acotiamide-resistant FD; two patients had a score of 1, and two patients had a score of 2. In contrast, two patients treated with vonoprazan alone had a score of 0 on EUS, four patients had a score of 1, and four patients had a score of 2. Yamabe et al. [23] and Rajan et al. [24] reported that changes in EUS features are nonspecific and can be observed in healthy patients. Ikeda et al. [25] and Petrone et al. [26] demonstrated that advanced age is also significantly associated with an increased risk of MPD dilatation. Therefore, we also have to consider the estimation of EUS scores in the view of age and nonspecific EUS features. In our data, only trypsin levels in five pancreatic enzymes exhibited significant differences in the vonoprazan and acotiamide combination group between improved and unimproved patients. We have previously reported that visceral hypersensitivity-related protease-activated receptor-2 (PAR-2) as the receptor for trypsin was widely expressed in the duodenum of FD patients with pancreatic enzyme abnormalities [27,28]. We speculate that trypsin-PAR-2 activation will lead to duodenal inflammation in patients with unimproved FD. Further studies will be needed to clarify why only trypsin levels in the unimproved group will be significantly higher than those in the improved group.

This study has some limitations. First, this was a small-sized study and the enrollment was undertaken in two institutions. Only 42 patients with vonoprazan and 31 patients with vonoprazan and acotiamide were enrolled. Second, we classified patients with FD based on Rome III criteria. Third, although we estimated serum elastase-1 and serum trypsin in this study, stool elastase-1 is more accurate than serum elastase-1 [29] and suggestive of pancreatic insufficiency and serum trypsinogen is more accurate than serum trypsin [30].

Taken together, the ratio of pancreatic enzyme abnormalities in unimproved patients treated with the combination of vonoprazan and acotiamide was significantly different from those in improved patients treated with combination therapy.

## Conclusions

Long-term vonoprazan alone or vonoprazan and acotiamide combination therapy significantly improved each FD symptom. The overall treatment efficacy in patients treated with vonoprazan alone or vonoprazan and acotiamide combination therapy was only 50%. In addition, there was a significant difference in the ratio of pancreatic enzyme abnormalities between improved and unimproved patients treated with vonoprazan and acotiamide combination therapy.
